# Supplementary material for: Increased ACS Enzyme Dosage Causes Initiation of Climacteric Ethylene Production in Tomato
Source: Int J Mol Sci. 2022 Sep 15;23(18):10788. doi: 10.3390/ijms231810788 (PMC9501751; doi:10.3390/ijms231810788)
Supplement: Supplementary file 1 [file ijms-23-10788-s001.zip › ijms-1892158-supplementary.pdf]

Table S1. List of primers used in this study

Target gene	Primer name	Sequence (5' - 3')	Reference
**For qPCR**			
SlUbiquitin	SlUbiquitin-F	CACCAAGCCAAAGAAGATCA	Takayama et al., 2015
	SlUbiquitin-R	TCAGCATTAGGGCACTCCTT	
LEACS1A	ACS1A-F	GACTTAAAGAAGGGGGAAAATGGTA	Shinozaki et al., 2015
	ACS1A-R	TCACTCATCACTACAACAAAAGGAA	
LEACS2	ACS2-F	GGAGGTTCGTAGGTGTTGAG	Mantelin et al., 2013
	ACS2-R	TAATGGTGAGGGAGGAATAGGT	
LEACS4	ACS4-F	AACAAGCACAATGGAAGAGGA	Mantelin et al., 2013
	ACS4-R	CGCACTACGAGCAAGGAAT	
LEACS6	ACS6-F	CAACAACCCAAAAGCCTCAAT	Wang et al., 2018
	ACS6-R	CTCCTGTTCTCCATCTCAAATCTCT	
LEACO1	ACO1-F	ACCATGTCCTAAGCCCGATTT	Shinozaki et al., 2015
	ACO1-R	ATTCGTGTCCCGTCTGTTTGT	
LEACO3	ACO3-F	CATGTCCTAAGCCCGATTTGA	Shinozaki et al., 2015
	ACO3-R	CCGAGTCCCATCTGTTTGTG	
NOR	NOR-F	TACCGGAACCGACAAGC	This study
	NOR-R	ATATCATGCAAATCATCTATGGACC	
RIN	RIN-F	CATGGCATTGTGGTGAGCAAAGTGT	Wang et al., 2018
	RIN-R	AGCATCATGTGTTGATGGTGCTGC	
PSY1	PSY1-F	CTGAGATCTACCAATGAGTTAG	Efremov et al., 2020
	PSY1-R	TCTCGGGAGTCATTAGCATAG	
ZDS	ZDS-F	TGCTTCAATGTGTCCTTACACC	This study
	ZDS-R	TGACCAGGTAACCTCAAGACCT	
LCYE	LCYE-F	ACAGGTTATTCAGTCGTCA	This study
	LCYE-R	CCAGTCCAAATAGGAAAAACGA	
LCYB	LCYB-F	TTGACTTAGAACCTCGTTATTGG	This study
	LCYB-R	AACAGTTCCCTTTGTCATTATCTC	
**For Sequencing & methylation sensitive RT-PCR**			
NOR-upsteam	*NOR*-upsteam-F	AAAACTAATTAAATCCGTCCA	This study
	NOR-upsteam-R	TGATAACTGAAAATTAGGCAA	

Table S2. TEs that identified in the regulatory sequence of *ACS4*

	Query		Subject			
	From	To	Name	From	To	Class
S. lycopersicum						
	−1866	−1735	BEL-24_TrVa-I	2118	2270	LTR/BEL
	−1244	−1151	Gypsy-36_BiGl-I	150	244	LTR/Gypsy
	−1150	−1115	Mariner-110_CCri	1564	1598	DNA/Mariner
	−1047	−912	CR1-49_HM	603	715	NonLTR/CR1
	−887	−772	Gypsy-22_GR-I	73	196	LTR/Gypsy
	−585	−397	EnSpm-6_VV	423	578	DNA/EnSpm/CACTA
S. lycopersicum var. *cerasiforme*						
	−1827	−1696	BEL-24_TrVa-I	2118	2270	LTR/BEL
	−1205	−1113	Gypsy-36_BiGl-I	151	244	LTR/Gypsy
	−1112	−1076	Mariner-110_CCri	1563	1598	DNA/Mariner
	−1074	−907	MUDRAVI1	11199	11364	DNA/MuDR
	−576	−517	Gypsy13-I_SP	6204	6262	LTR/Gypsy
	−515	−328	HARB-N23_ALy	262	455	DNA/Harbinger
S. pimpinellifolium						
	−1813	−1682	BEL-24_TrVa-I	2118	2270	LTR/BEL
	−1191	−1098	Gypsy-36_BiGl-I	150	244	LTR/Gypsy
	−1097	−1062	Mariner-110_CCri	1564	1598	DNA/Mariner
	−1061	−964	hAT-19B_ArHy	4938	5023	DNA/hAT
	−808	−671	Zator-9_RIr	3965	4123	DNA/Zator
	−584	−397	EnSpm-6_VV	423	578	DNA/EnSpm/CACTA
S. pennellii						
	−1805	−1760	Gypsy-103_ALy-I	2839	2882	LTR/Gypsy
	−1377	−1244	BEL-24_TrVa-I	2051	2211	LTR/BEL
	−1066	−1016	CR1-20_CGi	269	318	NonLTR/CR1
	−845	−724	hAT-26_HM	2927	3058	DNA/hAT
	−716	−661	hAT-7_HM	1715	1765	DNA/hAT
	−528	−394	EnSpm-6_VV	467	582	DNA/EnSpm/CACTA

Table S3. TEs that identified in the regulatory sequence of *LCYE*

	Query		Subject			
	From	To	Name	From	To	Class
S. lycopersicum	−1990	−1942	hAT-N5_NS	686	735	DNA/hAT
	−1711	−1635	TRAS-1_PXu	3703	3773	NonLTR/R1
	−1020	−723	MuDR-N2_SLy	1	304	DNA/MuDR
	−679	−396	hAT-N9_STu	1	281	DNA/hAT
	−56	−14	HARB-5_OS	2080	2122	DNA/Harbinger
S. lycopersicum var. *cerasiforme*						
	−1991	−1943	hAT-N5_NS	686	735	DNA/hAT
	−1712	−1636	TRAS-1_PXu	3703	3773	NonLTR/R1
	−1021	−723	MuDR-N2_SLy	1	304	DNA/MuDR
	−679	−396	hAT-N9_STu	1	281	DNA/hAT
	−56	−14	HARB-5_OS	2080	2122	DNA/Harbinger
S. pimpinellifolium						
	−1990	−1942	hAT-N5_NS	686	735	DNA/hAT
	−1721	−1618	Daphne-97_HMa	356	453	NonLTR/Daphne
	−1403	−1173	MuDR-14_GM	291	512	DNA/MuDR
	−1167	−1060	Harbinger-N2_NS	200	301	DNA/Harbinger
	−1021	−722	MuDR-N2_SLy	1	304	DNA/MuDR
	−678	−395	hAT-N9_STu	1	281	DNA/hAT
	−357	−216	MuDR-N7_AT	380	511	DNA/MuDR
	−56	−14	HARB-5_OS	2080	2122	DNA/Harbinger
S. pennellii						
	−1997	−1737	MuDR-N2_SLy	1	264	DNA/MuDR
	−1692	−1410	hAT-N9_STu	1	281	DNA/hAT
	−1149	−771	MuDR-8_STu	5779	6156	DNA/MuDR
	−370	−144	MuDR-8_STu	1	230	DNA/MuDR

Table S4. Comparison table for ethylene biosynthesis systems in tomato species

	S. lycopersicum cv. Ailsa Craig	*S. pimpinellifolium*	*S. pennellii*	*S. peruvianum*
ACS genes	*LEACS1A*			
	LEACS2	*LEACS2*		
	LEACS4	*LEACS4*	*LEACS4*	
	LEACS6	*LEACS6*	*LEACS6*	*LEACS6*
ACO genes	*LEACO1*	*LEACO1*	*LEACO1*	*LEACO1*
	LEACO3	*LEACO3*	*LEACO3*	*LEACO3*
Transcription factors	*NOR*	*NOR*		
	RIN	*RIN*		
Ethylene-induced *LEACS2* expression	✓	✓	✓	X
Ethylene-repressed *LEACS6* expression	✓	✓	✓	✓
Demethylated *NOR* promoter	✓	✓	✓	-

Expression levels for every gene across all wild species are compared with Ailsa Craig.
The lowly expressed genes are excluded form comparison table, which are 10 times lower than that of Alisa Craig.

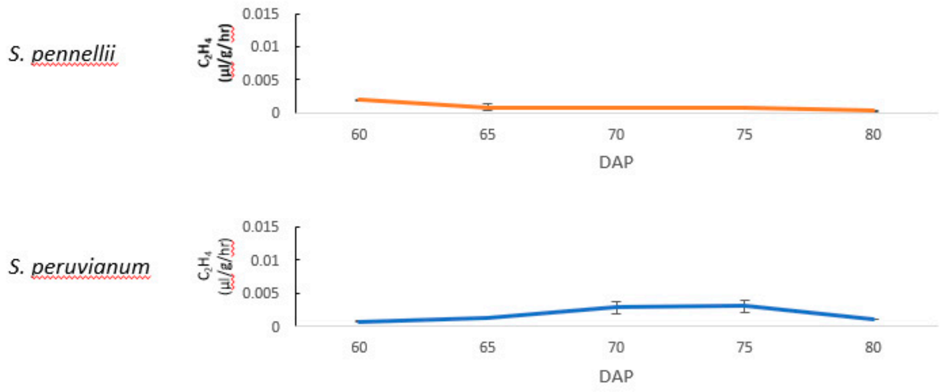

Figure S1. Quantification of ethylene production from 60 to 80 DAP. DAP: day after pollination. Fruits were observed from 60 to 80 DAP for *S. pennellii* and *S. peruvianum.*

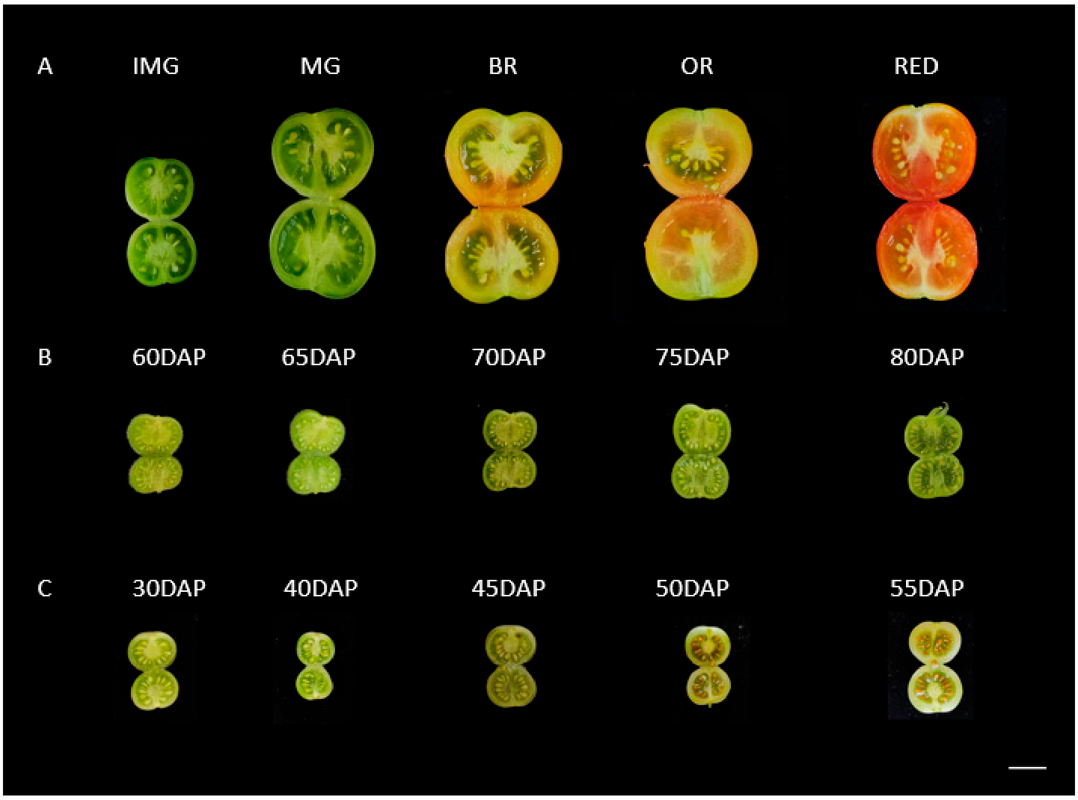

Figure S2. Seed maturation in green-ripe wild relatives. Ripening process and seed maturation for *S. lycopersicum* cv. Ailsa Craig (A), *S. pennellii* (B) and *S. peruvianum* (C). Scale bar = 1 cm. DAP: day after pollination. IMG: immature green; MG: mature green; BR: breaker; OR: orange; RED: red.

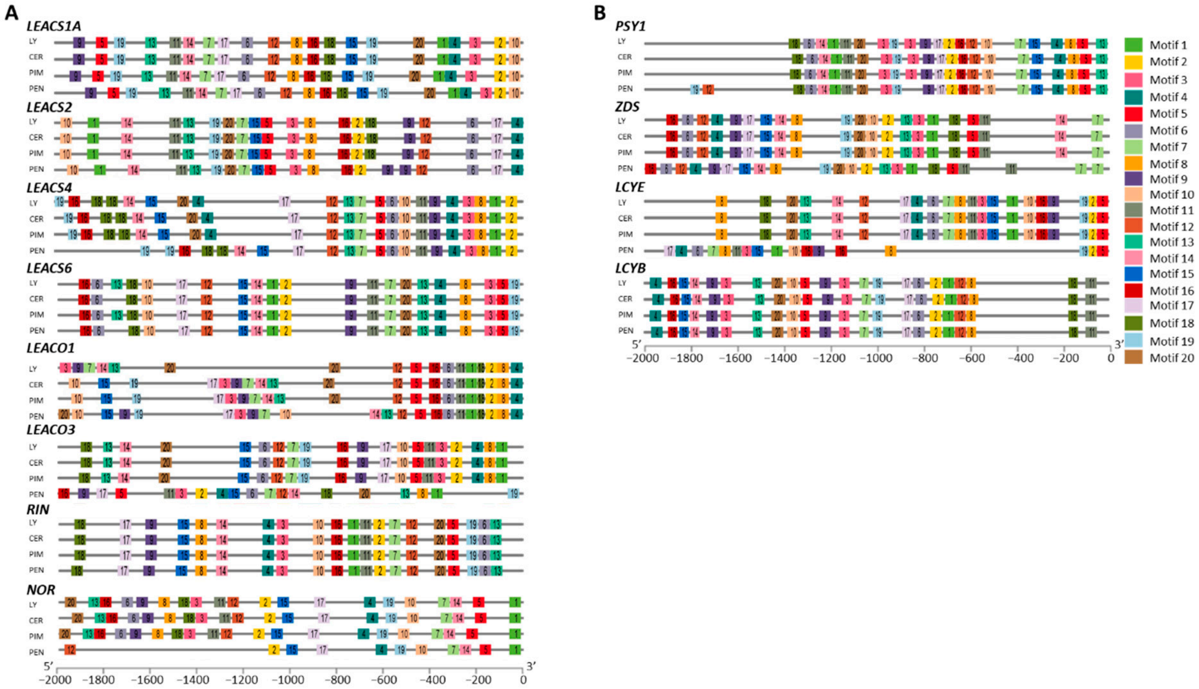

Figure S3. ***In silico* analysis of gene promoter regions.** MEME motif analysis of the regulatory sequence in ethylene biosynthetic genes (A). MEME motif analysis of the regulatory sequence in carotenoid biosynthesis-related genes (B). Each motif is represented by a colored box with a corresponding number. The order of the motifs correspond to the position in individual upstream sequences. The number of motifs does not correspond to the same motif among different genes. LY: *S. lycopersicum*; CER: *S. lycopersicum* var. *cerasiforme*; PIM: *S. pimpinellifolium*; PEN: *S. pennellii*.

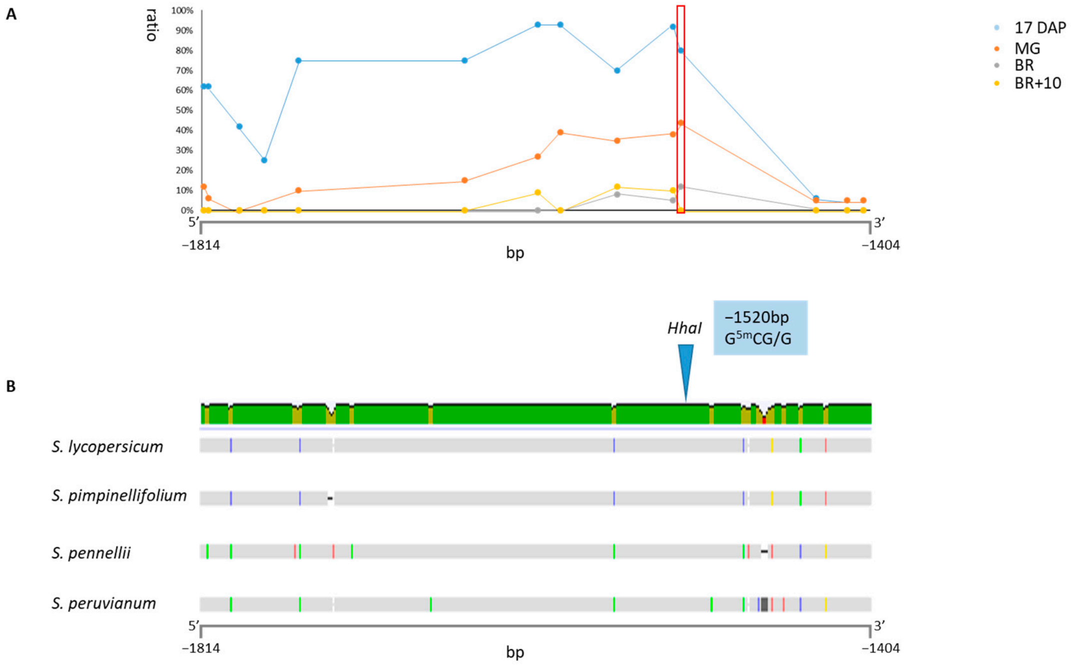

Figure S4. Upstream region of *NOR* for DNA methylation assay. Methylation level of a conservative region (−1814bp to −1414bp) in the upstream of *NOR* in *S. lycopersicum* (A). Data downloaded from tomato epigenome database (http://ted.bti.cornell.edu/epigenome/). Position of SNPs (B). Red indicated adenine, green indicated thymine, yellow indicated guanine, blue indicated cytosine and dark grey indicated unknown.

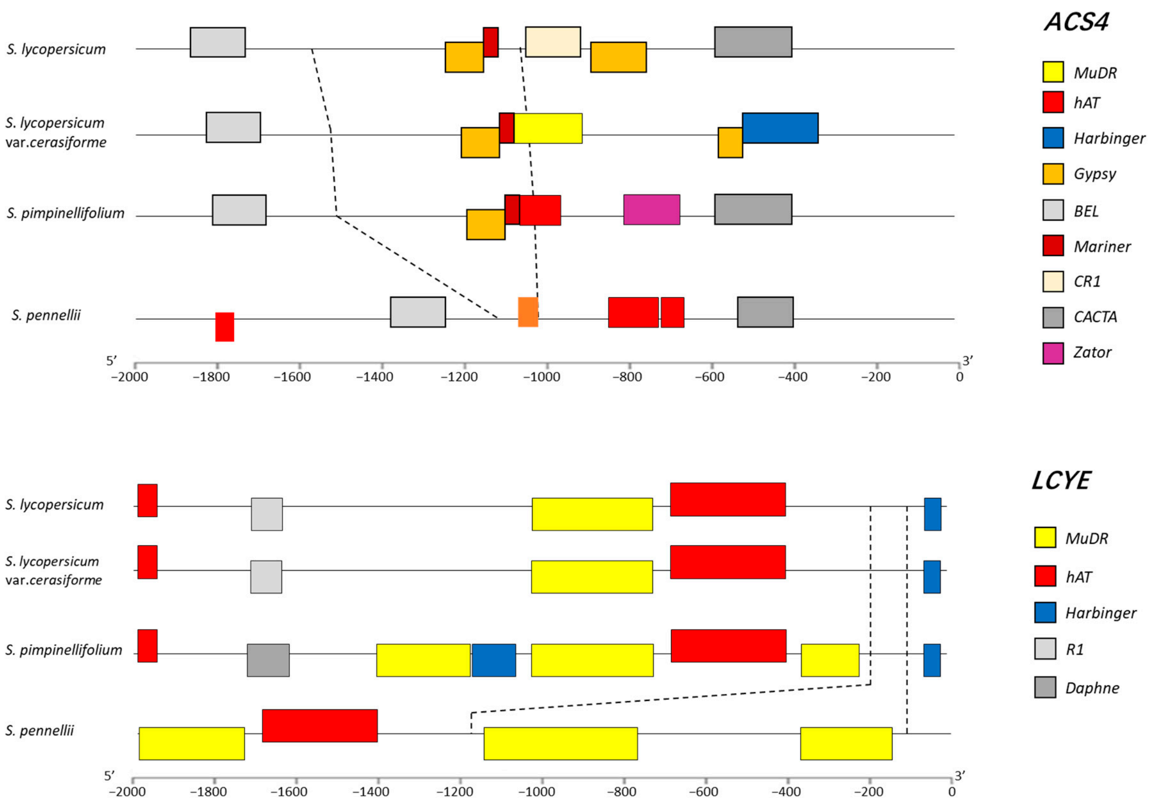

Figure S5. TEs identified in the regulatory sequence of *ACS4* and *LCYE*. Break lines show the position and length of insertion in the regulatory sequences. Upper and lower box indicates orientation of repeat fragment. Upper, sequence in same orientation as the locus; lower, sequence in opposite orientation.
